# Two-Dimensional GC–ToFMS Analysis of Volatile Organic Compounds in Fermented Camel Milk (Shubat)

**DOI:** 10.3390/foods14172995

**Published:** 2025-08-27

**Authors:** Sagyman Zhadyra, Fei Tao, Ping Xu

**Affiliations:** 1 State Key Laboratory of Microbial Metabolism, School of Life Sciences and Biotechnology, Shanghai Jiao Tong University, Shanghai 200240, China; sagyman@alumni.sjtu.edu.cn (S.Z.); pingxu@sjtu.edu.cn (P.X.); 2 Laboratory of Biotechnology, Research Institute for Biotechnology and Ecology, Zhetysu University, Taldykorgan 040009, Kazakhstan

**Keywords:** Shubat fermented camel milk, volatile organic compounds, GC×GC–TOF-MS, HS-SPME, metabolomics

## Abstract

Shubat, a traditional fermented camel milk from Kazakhstan, is renowned for its unique flavor and nutritional properties, though its volatile compound profile remains poorly characterized. In this study, headspace solid-phase microextraction coupled with comprehensive two-dimensional gas chromatography–time-of-flight mass spectrometry (HS-SPME-GC×GC–ToFMS) was employed to qualitatively identify and semi-quantitatively analyze volatile metabolites in seven Shubat samples collected from four regions of Kazakhstan. Of the 372 volatile organic compounds initially detected, 202 were retained after screening, predominantly comprising esters, acids, alcohols, ketones, and aldehydes. Esters, acids, and alcohol were found to be the most abundant categories. Diversity analyses (α and β) revealed substantial variation across regions, likely influenced by Shubat’s rich and region-specific microbiome. An UpSet analysis demonstrated that 75 volatile compounds were shared among all samples, accounting for over 87% of the total volatile content, indicating a chemically stable core. These findings underscore the chemical complexity of Shubat and provide novel insights into its metabolite composition, thereby establishing a foundation for future sensory, microbial, and quality-related research.

## 1. Introduction

Shubat is traditionally produced by fermenting fresh raw camel milk with a small amount of previously soured milk starter culture (~50 mL/L) in goat skin bags or wooden containers for 1–2 days [1]. During fermentation, the product is continuously or intermittently stirred to ensure sufficient aeration and uniformity. Owing to its long history of consumption among Kazakh nomads, Shubat is widely recognized for its high nutritional value and health-promoting properties. Shubat shares similarities with other fermented camel milk products, which are increasingly recognized for their functional properties [2,3]. For instance, fermented camel milk has been reported to possess anti-inflammatory, antidiabetic, lipid-lowering, and antimicrobial activities, attributed to its unique bioactive components, including organic acids and peptides. Moreover, short-chain fatty acids (SCFAs) such as acetic, propionic, and butyric acids, commonly produced through microbial fermentation, are known to play key roles in gut health, the modulation of immune function, and intestinal barrier integrity [4]. These properties suggest that the volatile organic compounds detected in Shubat may contribute to its health benefits and support its potential as a functional beverage.

Metabolism plays a central role in shaping the phenotypic characteristics of all living organisms, where low-molecular-weight biochemical compounds (metabolites) serve as intermediate and end products of enzymatically catalyzed reactions within cells [5]. These metabolites, often modulated by genetic and environmental factors, provide critical insights into cellular functions and physiological states. Following the emergence of genomics, transcriptomics, and proteomics, “metabolomics” has recently become an essential high throughput “omics” platform that enables the systematic study of the metabolic profile of organisms. It is particularly valuable for revealing biochemical mechanisms underlying food composition, fermentation, and flavor development [6,7].

In the context of fermented dairy products, microbial diversity and its associated metabolic pathways have a profound impact on final flavor, texture, and quality [8]. The characteristic flavor of fermented milk products is primarily attributed to volatile organic compounds (VOCs), such as esters, alcohols, acids, aldehydes, and ketones, many of which are generated through microbial fermentation processes [9]. Given their sensory importance, the identification and quantification of VOCs in complex food matrices has become a central focus in food science, as VOCs are often responsible for desirable aromas and product differentiation across cultures and regions.

Various analytical strategies have been developed for VOC profiling, among which headspace solid-phase microextraction (HS-SPME) has proven to be an efficient, solvent-free, and highly sensitive sample preparation method. First introduced by Pawliszyn et al. in 1989 [10], HS-SPME relies on the partitioning of analytes between a coated fiber and the sample matrix under equilibrium conditions. It can be coupled with gas chromatography (GC) for the separation and detection of volatile compounds. Fiber selection, extraction conditions, and analyte properties significantly affect the efficiency of this technique [11].

However, due to the complexity and diversity of food metabolites, conventional one-dimensional (1D) GC-MS methods often suffer from co-elution and limited resolution, restricting comprehensive VOC characterization. To overcome these challenges, the comprehensive two-dimensional gas chromatography–time-of-flight mass spectrometry (GC×GC–ToFMS) technique has emerged as a powerful tool, offering improved separation efficiency, structured chromatographic space, enhanced sensitivity, and the ability to capture low-abundance compounds that contribute significantly to flavor perception [12,13]. This technique has shown excellent performance in analyzing VOCs in complex matrices such as environmental samples, oils, and fermented foods [14].

From a molecular perspective, fermented dairy products, such as Shubat, represent highly dynamic chemical systems composed of proteins, lipids, amino acids, organic acids, carbohydrates, and aromatic volatiles [15]. Accurately profiling the volatile metabolome of Shubat requires integrative analytical platforms capable of capturing this diversity. GC×GC–ToFMS, when combined with optimized sample preparation such as HS-SPME, enables the high-resolution detection of a wide spectrum of volatile compounds, providing a comprehensive metabolic fingerprint of Shubat.

Recent research, including our previous metagenomic analysis of Shubat [16], has demonstrated that the rich microbial diversity in fermented camel milk plays a vital role in modulating its flavor-associated metabolic profile. These microbial communities, composed of diverse lactic acid bacteria (LAB) and yeast, participate in the biosynthesis of esters, alcohols, and acids that define the sensory identity of the product. Yet, the volatile metabolomic composition of Shubat remains underexplored.

Therefore, in this study, we applied headspace solid-phase microextraction coupled with comprehensive two-dimensional gas chromatography–time-of-flight mass spectrometry (HS-SPME-GC×GC–ToFMS) to comprehensively profile and classify volatile organic compounds (VOCs) in Shubat samples collected from different locations in Kazakhstan. This high-resolution technique offers enhanced separation efficiency and sensitivity compared to conventional GC–MS, enabling the accurate characterization of trace-level compounds in complex dairy matrices. The aim of this pilot study was to characterize the major classes of VOCs present in Shubat and to explore compositional differences among samples from distinct collection sites.

## 2. Materials and Methods

### 2.1. Sample Collection

Seven fermented camel milk (Shubat) samples were obtained from rural households located across four provinces of Kazakhstan: Almaty (S1, S2), Kyzylorda (S3), Zhetisu (S4, S7), and Turkestan (S5, S6). Each representative sample was formed by pooling three individual subsamples, which were separately sourced from different families residing in the same village. Although certain samples were sourced from the same administrative region, they were derived from geographically isolated villages, thus maintaining the independence of the samples.

After collection, the subsamples were carefully homogenized, and 50 mL of each pooled sample was transferred into sterile polypropylene tubes using aseptic c techniques. The samples were then pre-cooled, packed in insulated containers with ice packs, and immediately transported to the laboratory, where they were stored at −20 °C until subsequent analysis.

### 2.2. Volatile Metabolite Analysis

#### 2.2.1. Headspace Solid-Phase Microextraction (HS-SPME)

Frozen Shubat samples were thawed at room temperature, and 5 mL of the samples were transferred into a 15 mL glass vial. The vial was sealed with a PTFE/silicone septum and magnetic screw cap (Agilent Technologies, Santa Clara, CA, USA), followed by equilibration at 55 °C for 30 min. A 1 cm DVB/CAR/PDMS fiber (Supelco) (Merck KGaA, Darmstadt, Germany) was then inserted into the vial headspace for extraction. The sample was pre-incubated at 55 °C for 10 min, followed by extraction for 50 min. After extraction, the fiber was desorbed in the GC injection port for 5 min, and then thermally cleaned at 290 °C for 10 min to avoid cross-contamination.

#### 2.2.2. GC×GC–ToFMS Analysis

Volatile compounds were analyzed using a GC×GC–ToFMS system, consisting of an Agilent 7890 gas chromatograph (Agilent Technologies, Santa Clara, CA, USA) coupled with a Pegasus 4D time-of-flight mass spectrometer (LECO Corporation, St. Joseph, MI, USA). The analysis parameters were as follows: Primary column: DB-WAX (30 m × 250 μm × 0.25 μm; Agilent Technologies, Santa Clara, CA, USA). Secondary column: DB-17MS (2 m × 100 μm × 0.10 μm; Agilent Technologies, Santa Clara, CA, USA), Injection temperature: 250 °C. Carrier gas: Helium (99.99%) at 1.0 mL/min (constant flow, splitless mode). Oven temperature program: 40 °C for 5 min; ramped at 5 °C/min to 140 °C (held 2 min); then 10 °C/min to 250 °C (held 12 min). Secondary column oven: 15 °C higher than the primary column. Modulator temperature: 15 °C higher than the secondary column. Modulation period: 5.0 s. Transfer line temperature: 270 °C. Ion source temperature: 230 °C. Ionization mode: Electron impact (EI), 70 eV. Detector voltage: 1670 V. Mass range: *m*/*z* 33–550. Acquisition rate: 50 spectra/s.

### 2.3. Data Preprocessing, Compound Identification, and Visualization

Raw chromatographic data were processed using LECO ChromaTOF^®^ software (version 4.72.0.0), which performed automated baseline correction, peak deconvolution, alignment, and noise reduction. Primary and secondary dimension peak widths were set to 16 s and 0.2 s, respectively.

To eliminate non-sample-related signals, procedural blanks were analyzed under identical conditions, and all peaks detected in blanks were excluded from the dataset. Retention indices (RIs) were determined using a C5–C40 n-alkane standard mixture and calculated via the Kovats index method. Mass spectral identification was conducted using the NIST 17 Mass Spectral Library, and only peaks with forward and reverse match scores above 700 were retained [17]. The calculated RI values were compared to literature data (e.g., NIST Chemistry WebBook), and compounds with deviations exceeding ±5% were excluded. Identification confidence corresponds to Level 2 of the Metabolomics Standards Initiative (MSI), indicating putative annotation based on spectral and RI matching without validation by authentic standards. To ensure data robustness, peaks containing halogen or silicon atoms were removed to avoid potential artifacts from column bleed or contamination. Additionally, low-frequency compounds, defined as those detected in fewer than four of the seven Shubat samples, were excluded prior to statistical analysis.

Volatile compound abundance matrices were generated after data preprocessing, which included removing contaminants, compounds detected in blank samples, and non-organic compounds. Peak areas of each compound were converted to relative abundances within each sample. To further focus on the major contributing compounds and enhance the interpretability of the results, the normalized dataset was ranked based on the total abundance of each compound across all samples. The top 50 most abundant volatile compounds were selected for subsequent statistical analysis and visualized in tabular form as percentage values relative to the total volatile profile. 

To investigate the overlap and uniqueness of volatile compounds across different Shubat samples, a presence/absence matrix was constructed based on the qualitative identification results. In this binary matrix, the presence of a compound in each sample was encoded as 1 and absence was encoded as 0. The binary data were then analyzed using the upsetplot (v0.8.0). An UpSet plot was generated to visualize the number of shared and unique compounds among the seven Shubat samples. 

Alpha diversity indices were calculated using Python (v3.12) using the pandas and scikit-bio packages. Beta diversity was assessed using Bray–Curtis dissimilarity based on relative abundance data, and principal coordinate analysis (PCoA) was performed using scikit-bio. Data visualization, including heatmaps and PCoA plots, was carried out using matplotlib and seaborn. Due to logistical and field constraints, only one representative Shubat sample was collected from each village across four regions. Replicates or composite samples were not available for each region. Therefore, the diversity analyses presented in this study are exploratory in nature and do not allow for the statistical validation of intra-regional variability.

## 3. Results

### 3.1. Major Volatile Compound Categories in Shubat Samples

**Figure 1:**
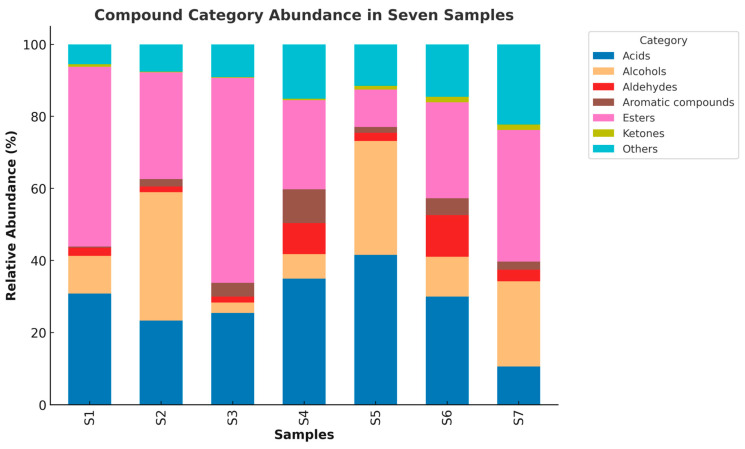
Stacked bar plot illustrating the relative abundance of major volatile compound categories across seven Shubat samples (S1–S7).

To characterize the VOCs, present in the seven Shubat samples we performed qualitative identification and relative quantification using headspace solid-phase microextraction coupled with GC×GC–TOF-MS. A total of 372 volatile organic compounds (VOCs) were detected in Shubat samples collected from four regions of Kazakhstan, of which 202 were retained for compositional analysis. These VOCs were primarily classified into six major categories: esters, acids, alcohols, ketones, aldehydes, and aromatic compounds. The relative abundance of each category varied across the seven samples (Figure 1), with detailed 3D chromatograms for individual samples provided in Supplementary Figure S1. Esters and acids were generally the dominant compound classes, though the proportion of alcohol and aldehydes showed notable variation among samples. Aromatic compounds were present in lower abundance but showed marked enrichment in S4 and S6.

Overall, the compositional differences in VOC categories across samples highlight the influence of regional origin, household fermentation practices, and microbiota composition on the volatile metabolome of Shubat.

A total of 50 volatile organic compounds (VOCs) with the highest average relative abundance were identified across the seven Shubat samples and are listed in Table 1. The detailed information for each sample is provided in Supplementary Table S1 (S1_top50–S7_top50), which includes retention indices, CAS numbers, and spectral match scores. Most of these VOCs belonged to esters, organic acids, and alcohols. Among them, acetic acid and ethyl acetate were the most abundant overall, with mean relative abundances of 19.60% and 15.27%, respectively. Acetic acid showed particularly high levels in samples S4, S5, and S6, whereas ethyl acetate was dominant in S1 and S6.

Ethanol, another major fermentation product, exhibited large variation among samples, with concentrations ranging from 0.00% to 36.28%. Medium-chain fatty acid ethyl esters such as ethyl decanoate, ethyl dodecanoate, and ethyl octanoate were also prominent, with the highest levels observed in samples S2 and S3. These esters are typically associated with fruity and creamy flavor characteristics in fermented dairy products.

Several volatile organic acids, including hexanoic acid, octanoic acid, and dodecanoic acid, were consistently detected across all samples. These acids contribute sour, pungent, or goaty notes depending on concentration and matrix interaction. Additionally, aromatic compounds such as benzaldehyde and 2-phenylethyl acetate were identified in moderate abundance, with the latter reaching 14.05% in sample S7, indicating sample-specific enrichment.

### 3.2. Core and Unique Volatile Compounds Across Shubat Samples

**Figure 2:**
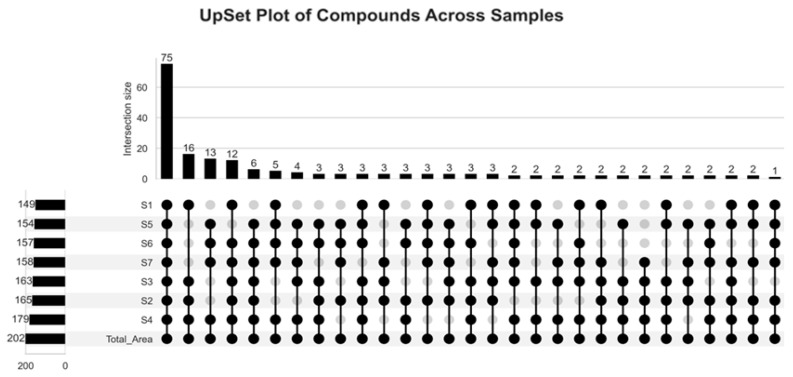
Visualization of Core and Sample-Specific Volatile Compounds in Shubat.

To explore the overlap and uniqueness of volatile compounds among the seven Shubat samples, an UpSet plot was constructed. As shown in Figure 2, a total of 75 compounds were found to be shared by all seven samples. Although these shared compounds only account for a small fraction of all detected compounds, their abundance accounts for about 87% of the total abundance of the overall volatile components. This indicates that a core set of volatile metabolites dominates the flavor composition of Shubat, suggesting that despite differences in origin, there is a high degree of consistency between samples at the metabolic level. 

### 3.3. Alpha and Beta Diversity Analysis of Volatile Compounds

**Figure 3:**
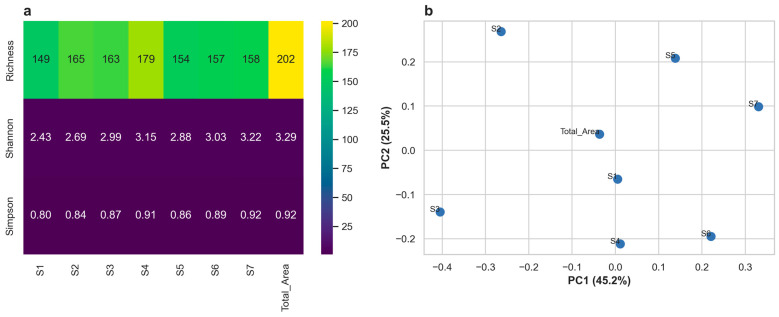
Diversity Analysis of Volatile Compounds in Shubat Samples. (**a**) Alpha diversity indices (Richness, Shannon, and Simpson) of volatile compounds across seven samples. (**b**) Beta diversity analysis based on Bray–Curtis dissimilarity visualized by PCoA.

The diversity of volatile organic compounds (VOCs) in the seven Shubat samples was evaluated using three alpha diversity indices: richness, Shannon, and Simpson. As shown in Figure 3a, richness ranged from 149 (S1) to 179 (S4), indicating moderate variation in the number of detected VOCs across samples. The Shannon index values ranged from 2.43 to 3.29, with the highest diversity observed in S7 and the lowest in S1, suggesting differences in both the number and evenness of VOCs. Simpson indices were relatively stable across the samples, ranging from 0.80 to 0.92, indicating a relatively even distribution of compounds with no single compound overwhelmingly dominant in any sample.

Beta diversity was assessed using Principal Component Analysis (PCA) based on VOC composition. The first two principal components explained 45.2% and 25.5% of the total variance, respectively (Figure 3b). The samples were clearly separated into the PCA space, reflecting substantial variation in volatile profiles among the different Shubat samples. No clear clustering pattern was observed in the PCA plot, indicating high inter-sample variability in volatile compound composition.

## 4. Discussion

In this study, we applied HS-SPME–GC×GC–TOF-MS to profile volatile organic compounds (VOCs) in Shubat samples, detecting a total of 372 compounds, which significantly exceeds the counts typically reported using conventional one-dimensional GC–MS methods [3,18]. This improved coverage reflects the enhanced separation performance and greater sensitivity afforded by the GC×GC platform.

Despite advances in deconvolution algorithms for one-dimensional GC–MS, co-elution remains a major challenge in complex matrices such as fermented dairy. In contrast, comprehensive two-dimensional GC delivers markedly improved peak capacity and selectivity, enabling better resolution of co-eluting compounds across two chromatographic dimensions (volatility and polarity) [19,20,21]. The modulation process in GC×GC refocuses analytes into sharp pulses, leading to substantially improved signal-to-noise ratios (S/N) and facilitates the detection of low-abundance metabolites that often go undetected in 1D GC–MS [22]. For Shubat, a highly complex fermented dairy matrix containing diverse proteins, lipids, and microbial metabolites, the use of GC×GC–TOFMS was particularly advantageous. This approach enabled the resolution of trace, aroma-relevant VOCs that would likely remain masked in conventional 1D analysis, thereby yielding a more reliable metabolic fingerprint specific to Shubat fermentation.Moreover, this study emphasizes the contribution of Shubat’s rich microbiome to the observed diversity of volatile compounds. According to our recent metagenomic investigation of Shubat [16], the product harbors a diverse and metabolically versatile microbial community, particularly LAB such as *Lactobacillus*, *Lactococcus*, and *Streptococcus*. These microbes are involved in amino acid metabolism, carbohydrate utilization, and secondary metabolite biosynthesis, which may collectively contribute to the unique and complex volatile profiles observed in Shubat. This microbial influence provides an important biochemical basis for Shubat’s distinctive flavor characteristics and potential functional properties.

Compared to previous findings, the volatile compound profile obtained in this study shares both commonalities and differences. For instance, Ning et al. [18] identified esters, alcohols, and acids as the dominant volatile classes in fermented milk products, which aligns with our results, where esters and acids exhibited the highest relative abundance. Similarly, Bilal et al. [3] reported the presence of key volatiles such as ethyl acetate, acetic acid, and 2,3-butanediol in traditional fermented dairy products compounds that were also found at notably high levels in the Shubat samples examined here.

In the present study, the volatile profiles of Shubat were predominantly composed of acids, esters, and alcohols, followed by aldehydes, aromatic compounds, ketones, and minor volatile classes. Such a composition aligns with previous findings that fermented dairy products, including camel milk derivatives, typically contain these compound classes as key aroma contributors [23,24]. Notably, the dominance of esters in Shubat underscores their important role in flavor development; their formation via microbial esterification and lipid metabolism during dairy fermentation is well documented [25].

The relative stability of acids across samples suggests that they form a consistent chemical backbone for Shubat’s aroma, whereas variation in esters and alcohols likely reflects microbial metabolic differences linked to fermentation conditions or raw milk characteristics. Lower-abundance volatiles such as aldehydes and ketones, though present in smaller quantities, may still impart distinctive sensory nuances. This pattern echoes broader reports in fermented dairy research where compound diversity contributes to flavor complexity [26].

Esters are among the most prevalent volatile constituents in dairy and fermented milk products [27,28]. At low concentrations, these compounds contribute pleasant fruity aromas to milk and its derivatives [29]. However, elevated levels of esters may result in undesirable fruity off-flavors in dairy products [30]. In the present study, over 90 distinct ester compounds were identified, representing the most abundant chemical class among all detected volatiles. It is hypothesized that the biosynthesis of ethyl acetate and ethyl lactate may primarily originate from the esterification of microbial fermentation metabolites, lactic acid and hexanoic acid, with ethanol. These short-chain ethyl esters are considered favorable contributors to the fruity flavor observed in Shubat samples [31,32].

Two major biochemical pathways, esterification and alcoholysis, are proposed to play key roles in the formation of ester compounds in dairy matrices. Esterification refers to the formation of esters from the reaction of free fatty acids (FFAs) (released during the lipolysis of triglycerides by lipases or esterases) with alcohols. Alcoholysis, in contrast, involves the transesterification of fatty acyl moieties from mono-, di-, or triacylglycerols or fatty acyl-CoA derivatives directly to alcohols generated through amino acid or carbohydrate metabolism. Both pathways are enzymatically catalyzed by esterases or lipases [33]. LAB has been shown to exhibit both esterase and lipase activities, contributing significantly to the biosynthesis of ethyl esters in fermented dairy products. Several studies have reported esterase activity in LAB strains [34,35,36]. These enzymes not only catalyze the hydrolysis of milk fat triglycerides to release FFAs but also participate in transesterification reactions, leading to the synthesis of esters from glycerides and alcohols [37]. 

One of the most abundant acids, acetic acid, may originate from the heterofermentation of glucose, a hydrolysis product of lactose. In addition, high-abundance acetic acid bacteria are capable of oxidizing ethanol to produce acetic acid. FFAs with carbon chains of four or more atoms are often derived from the lipolysis of milk fat, which is a key factor influencing the flavour profile of fermented dairy products [38]. Triglycerides, comprising a glycerol backbone esterified with three fatty acid chains, represent the primary form of fat in milk. FFAs are fatty acids not bound to glycerol molecules, and they play a central role in the flavour development of dairy products, particularly cheeses and fermented milk [39]. 

Free fatty acids (FFAs) in dairy systems can be released enzymatically by lipases and esterases, which are either endogenous to milk or produced by microorganisms during fermentation. However, acid generation is not limited to lipid breakdown. Streptococcus spp. can convert lactic acid into formate, acetaldehyde, ethanol, and acetic acid [32,40,41], whereas *Propionibacterium* spp. utilize lactate to form propionic acid, acetic acid, and CO_2_. Additionally, certain lactic acid bacteria (LAB) strains can synthesize volatile fatty acids through amino acid catabolism [33,42]. These organic acids not only significantly influence the sensory properties of fermented dairy products but also serve as essential precursors for the formation of other volatiles, such as esters, ketones, and alcohols. 

Alcohols represented the fourth most abundant category among the volatile constituents identified in Shubat. The most prominent alcohol detected was ethanol, which consistently showed the highest relative abundance across all samples. Ethanol is a well-known volatile compound commonly found in fermented dairy products, typically produced as a byproduct of microbial metabolism, especially by yeasts and certain lactic acid bacteria [31,33]. Its presence is indicative of active fermentation processes and contributes to the characteristic aroma of fermented milk. 

Alcohols such as phenylethyl alcohol (2-phenylethanol), 1-butanol, 3-methyl-, and other higher alcohols contribute floral and green sensory nuances, enhancing the aromatic complexity of Shubat. 2-Phenylethanol, present at relatively high levels, is well known for its floral and sweet aroma notes and makes an important contribution to the sensory complexity of Shubat. Aromatic alcohol 2-phenylethanol, with a distinct rose-like fragrance [43], is widely used in the food, cosmetic, and fragrance industries [44]. Microbial synthesis is considered an important route for producing natural 2-phenylethanol. Various microorganisms such as *Aspergillus niger*, *Saccharomyces vini*, *Torulopsis utilis*, *Kluyveromyces marxianus*, and *Microbacterium* spp. have been reported to generate 2-phenylethanol during fermentation [45,46,47]. In our previous study [16], metagenomic sequencing revealed a high relative abundance of *Kluyveromyces marxianus* in the Shubat samples, which may partially explain the presence of 2-phenylethanol.

Other identified alcohols such as 1-hexanol are also commonly observed volatile constituents in dairy products [24,48]. These alcohols can arise through several biochemical pathways, including the reduction of ketones or aldehydes, and the microbial catabolism of amino acids, lactose, lipids, and citric acid [31,49].

Diversity analysis revealed distinct regional differences in the volatile metabolite profiles of Shubat. Both α- and β-diversity metrics indicated considerable chemical variation among the seven samples, suggesting strong region-specific influences. However, due to the absence of replicate or composite samples within each region, intra-regional variation cannot be statistically assessed. As a result, diversity comparisons should be regarded as preliminary, and future studies incorporating biological replicates are needed to validate these findings.

To assess beta diversity, we employed Principal Coordinates Analysis (PCoA) based on Bray–Curtis dissimilarity derived from relative volatile metabolite abundances. PCoA is particularly advantageous for exploratory metabolomics studies with limited or non-existent replicates, as it does not assume data normality or homogeneity of variance and is capable of handling non-Euclidean distance measures. This method is widely used for visualizing compositional dissimilarities among samples in metabolomics and microbial profiling (e.g., Bray–Curtis PCoA) [50,51]. Importantly, studies have demonstrated that β-diversity metrics such as Bray–Curtis are highly sensitive in detecting differences, even with small sample sizes, whereas α-diversity metrics may not reveal these distinctions under the same conditions [52]. In this context, PCoA provided a robust, informative view of regional variation in Shubat volatile profiles, despite the lack of replication.

## 5. Conclusions

This study presents the first in-depth profiling of volatile organic compounds in traditionally fermented Shubat using HS-SPME-GC×GC–ToFMS. After rigorous data processing, contaminant removal, and retention index filtering, 202 high-confidence compounds were retained from an initial 372. Esters, acids, and alcohol were the most abundant classes, reflecting typical fermentation-derived metabolic activity.

Alpha diversity analyses revealed substantial chemical complexity across the samples, and β-diversity assessments indicated regional variation in volatile profiles, likely shaped by distinct household fermentation practices and microbial communities. Despite this heterogeneity, 75 core volatile compounds were shared across all samples and accounted for over 87% of the total volatile abundance, suggesting a chemically conserved backbone associated with traditional Shubat fermentation.

Several key metabolites such as acetic acid, ethanol, and ethyl acetate were abundant across most samples, underscoring their critical role in shaping sourness, aroma, and effervescence. The detection of medium-chain ethyl esters and higher alcohols further suggests active microbial esterification and amino acid metabolism during fermentation. These compounds are central to Shubat’s distinctive flavor and may serve as potential markers for quality and authenticity.

Limitations include the relatively small number of samples and the lack of direct sensory or functional validation. Future studies should combine chemical, microbial, and sensory data to better link volatile profiles with flavor and health-related properties.

## Figures and Tables

**Table 1:** Top 50 Most Abundant Volatile Compounds Detected in Shubat Samples (Based on Total Relative Abundance).

Compounds	Relative Abundance (%)						
	S1	S2	S3	S4	S5	S6	S7
Acetic acid	24.27	14.01	15.13	25.62	31.17	27.01	0.01
Ethanol	9.27	36.28	0.00	0.00	26.02	0.60	14.77
Ethyl Acetate	37.87	2.52	3.99	12.52	7.00	22.73	20.26
Decanoic acid, ethyl ester	0.45	6.52	32.85	1.64	0.17	0.59	0.50
Hexanoic acid	3.46	4.48	1.25	4.94	6.24	3.10	5.05
Dodecanoic acid, ethyl ester	3.42	10.09	2.47	1.91	0.25	0.81	0.73
Benzaldehyde	0.17	1.91	3.53	9.56	1.31	4.72	1.80
1-Butanol, 3-methyl-	2.02	1.40	1.29	7.99	0.73	7.42	2.12
Acetic acid, 2-phenylethyl ester	1.92	1.49	2.63	1.16	0.94	1.51	14.05
Octanoic acid, ethyl ester	2.48	3.47	3.01	4.47	0.29	0.84	0.79
ETHYL (S)-(-)-LACTATE	0.00	0.00	0.00	6.31	1.37	5.42	10.07
Phenylethyl Alcohol	0.88	0.51	2.19	4.64	1.27	3.49	4.01
Tetradecanoic acid, ethyl ester	1.45	1.55	2.65	2.29	0.36	0.79	0.76
Pentadecanoic acid, ethyl ester	1.15	1.62	4.26	0.13	0.00	0.00	0.00
Octanoic acid	0.92	1.04	0.49	1.85	2.08	1.16	1.64
Dodecanoic acid	1.00	1.25	2.02	1.47	0.27	0.19	0.27
Ethyl tridecanoate	0.76	1.79	2.76	0.09	0.00	0.00	0.00
Tetradecanoic acid	1.09	0.58	2.34	1.41	0.28	0.19	0.28
1-Hexanol	0.14	0.00	0.00	0.26	0.93	3.08	4.58
Hexadecanoic acid, ethyl ester	1.45	0.16	2.59	0.70	0.00	0.11	0.00
1,3,5,7-Cyclooctatetraene	0.00	1.57	2.38	0.00	0.06	0.07	0.00
n-Decanoic acid	1.18	0.60	1.01	0.95	0.41	0.29	0.36
Butanoic acid	0.25	0.88	1.58	0.39	0.79	0.24	0.44
2-Nonenal, (E)-	0.03	0.02	0.04	0.40	1.87	2.52	1.71
Hexanal	0.18	0.06	0.08	0.78	0.58	3.52	0.26
Furan, 2-ethyl-	0.04	0.02	0.05	0.30	2.43	0.88	1.83
Ethyl 9-decenoate	0.40	0.80	1.47	0.56	0.02	0.05	0.04
Heptanoic acid	0.07	0.08	0.15	0.14	2.21	0.57	1.36
Hexanoic acid, ethyl ester	0.39	0.21	1.04	1.74	0.04	0.18	0.15
(E)-9-Octadecenoic acid ethyl ester	0.54	0.77	1.07	0.23	0.00	0.07	0.03
Benzoic acid	0.09	0.62	1.00	0.87	0.12	0.09	0.13
Acetoin	0.02	0.01	0.01	0.12	1.76	0.18	2.10
Butanoic acid, ethyl ester	0.12	0.64	1.25	0.25	0.00	0.05	0.05
Furan, 2-pentyl-	0.06	0.04	0.04	0.29	0.93	1.12	1.36
Nonanal	0.05	0.04	0.06	0.32	1.04	0.79	0.92
E-11-Hexadecenoic acid, ethyl ester	0.08	1.01	0.37	0.02	0.03	0.14	0.13
Propanoic acid, 2-methyl-	0.26	0.04	0.07	0.04	1.54	0.13	0.54
1-Heptanol	0.00	0.00	0.01	0.14	0.45	0.98	1.79
n-Hexadecanoic acid	0.16	0.48	0.65	0.35	0.08	0.07	0.10
9-Decenoic acid	0.17	0.36	0.62	0.69	0.06	0.04	0.05
Benzene	0.14	0.15	0.27	0.45	0.38	0.47	0.64
Ethyl 9-hexadecenoate	0.75	0.10	0.17	0.72	0.03	0.13	0.11
2-Decenal, (Z)-	0.04	0.02	0.04	0.18	0.84	0.77	0.90
Butanoic acid, 3-methyl-	0.22	0.04	0.00	0.11	0.85	0.08	1.31
1-Penten-3-one	0.01	0.00	0.01	0.10	1.63	0.22	0.23
1-Propanol, 2-methyl-	0.23	0.16	0.05	0.05	0.09	1.08	0.47
Z-7-Tetradecenoic acid	0.17	0.37	0.60	0.20	0.00	0.00	0.00
2-Octenal, (E)-	0.02	0.01	0.02	0.31	0.49	0.96	0.31
2-Undecenal	0.04	0.03	0.05	0.14	0.55	0.34	0.75
1-Propanol	0.11	0.18	0.41	0.19	0.03	0.20	0.24

## Data Availability

The original contributions presented in this study are included in the article/Supplementary Material, further inquiries can be directed to the corresponding author.

## Supplementary Materials

The following supporting information can be downloaded at: https://www.mdpi.com/article/10.3390/foods14172995/s1, Figure S1: Representative 3D chromatograms of volatile compounds detected in Shubat samples S1–S7 using GC×GC–ToFMS. Table S1: Top 50 volatile organic compounds (VOCs) across all samples (S1–S7). Data are provided in an Excel file containing seven sheets (S1_top50–S7_top50), where each sheet corresponds to one Shubat sample.
