# Supplementary material for: Two-Dimensional GC–ToFMS Analysis of Volatile Organic Compounds in Fermented Camel Milk (Shubat)
Source: Foods. 2025 Aug 27;14(17):2995. doi: 10.3390/foods14172995 (PMC12427637; doi:10.3390/foods14172995)
Supplement: Supplementary file 1 [file foods-14-02995-s001.zip › foods-3787811-supplementary/Figure S1.pdf]

Supplementary materials:

Figure S1. Representative 3D chromatograms of volatile compounds detected in seven Shubat samples (S1–S7) using GC×GC–ToFMS.

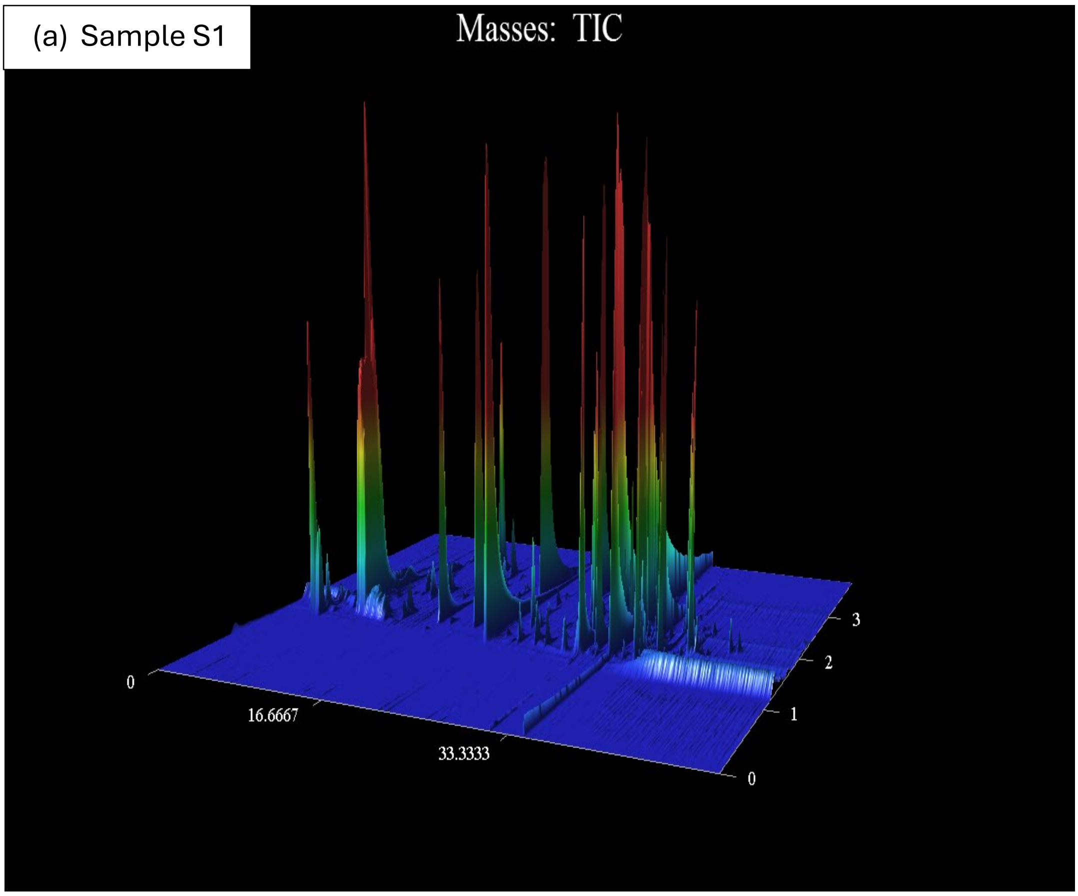

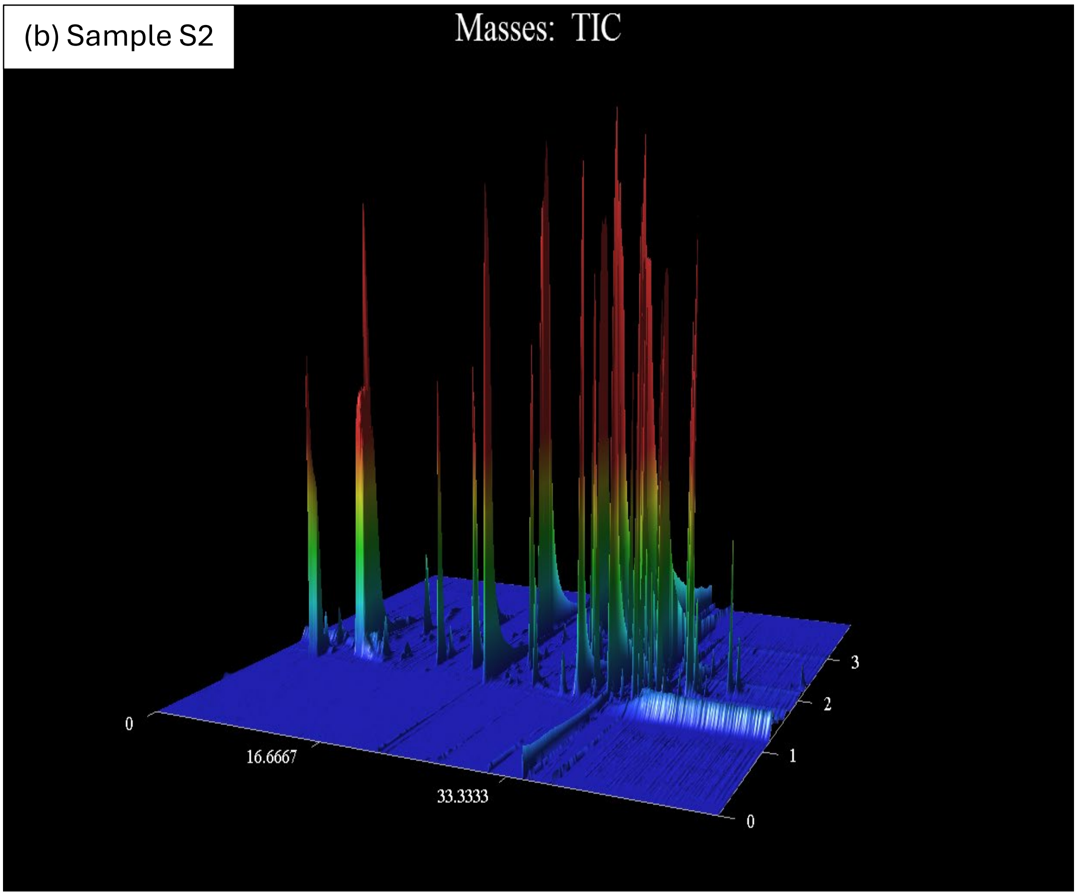

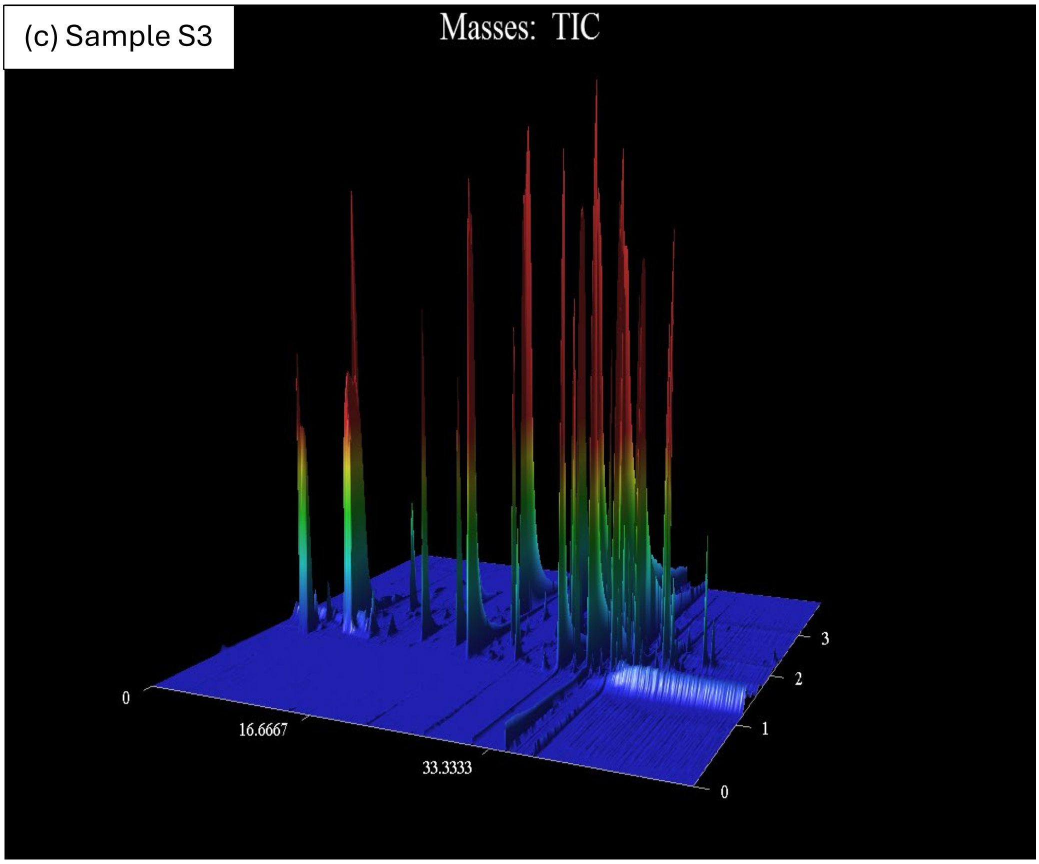
(c) Sample S3
Masses: TIC
0
16.6667
33.3333
0
1
2
3

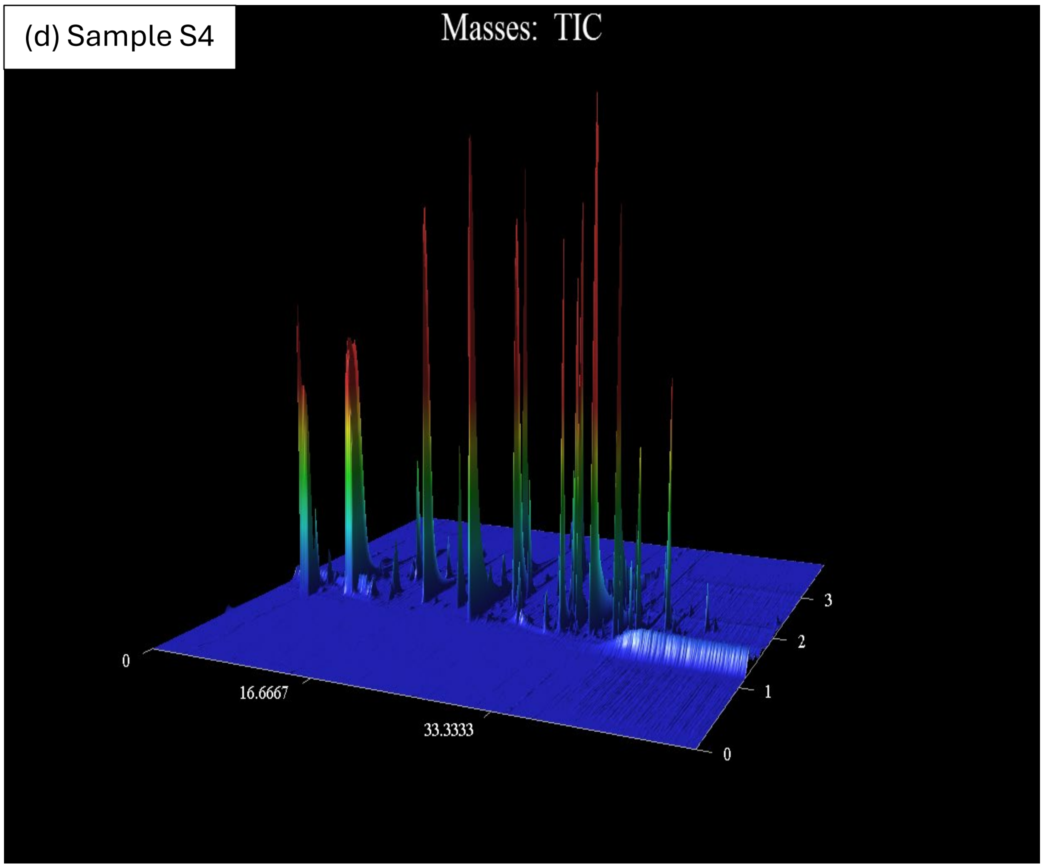
(d) Sample S4
Masses: TIC
0
16.6667
33.3333
0
1
2
3

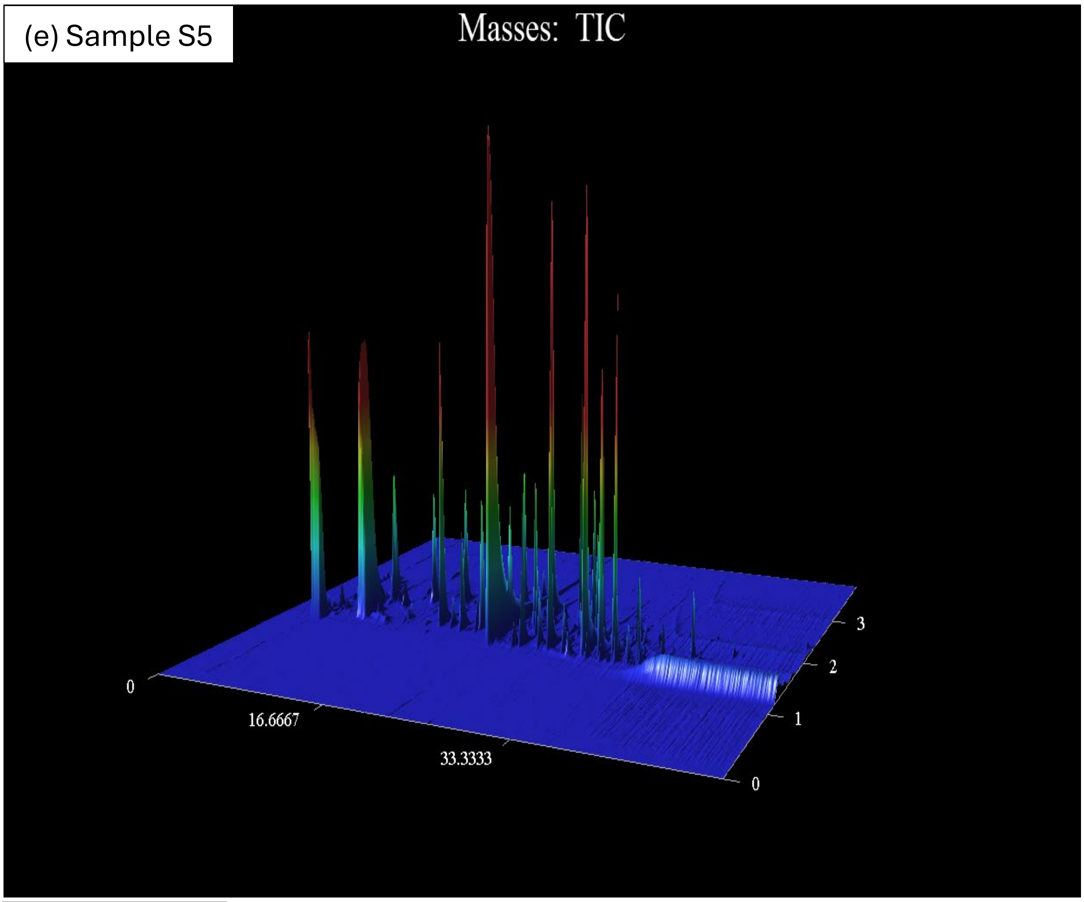
(e) Sample S5
Masses: TIC
0
16.6667
33.3333
3
2
1
0

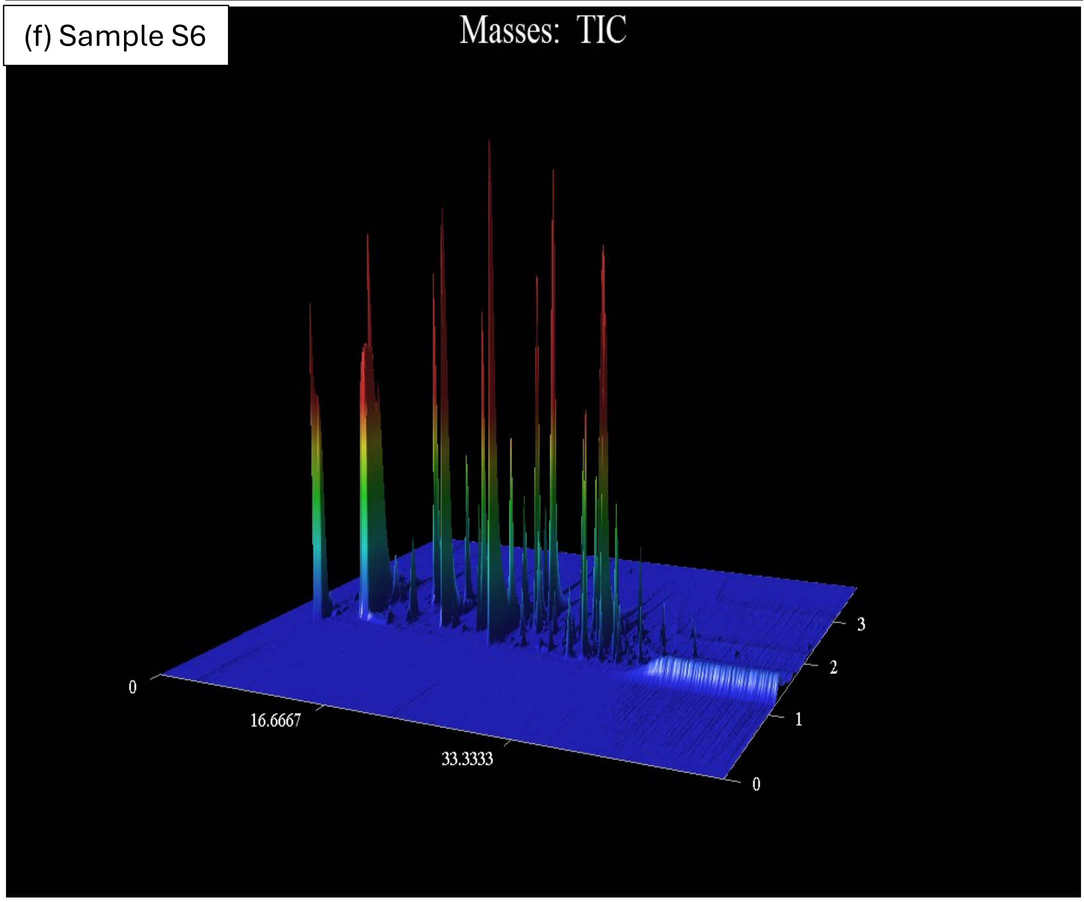
(f) Sample S6
Masses: TIC
0
16.6667
33.3333
3
2
1
0

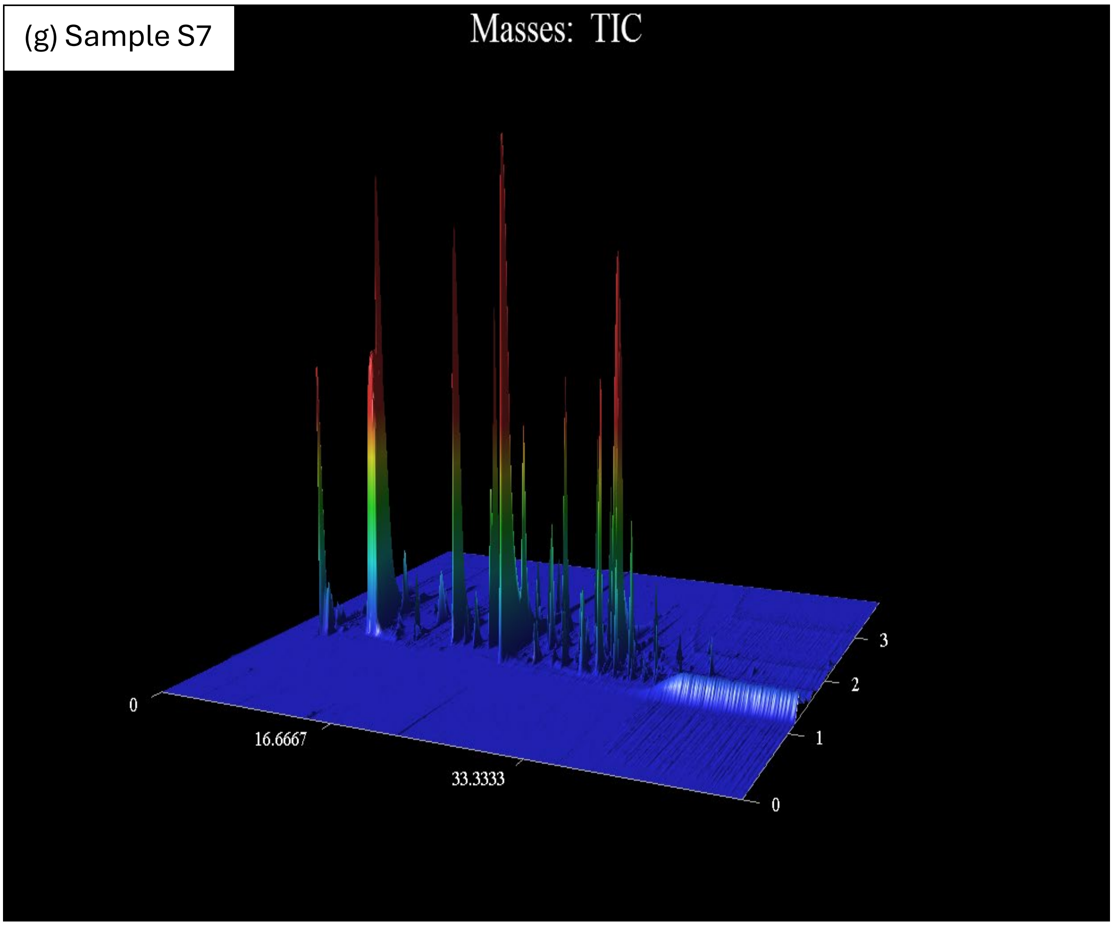
(g) Sample S7
Masses: TIC
0
16.6667
33.3333
0
1
2
3
